# Why We Need Minimum Basic Requirements in Science for Acupuncture Education

**DOI:** 10.3390/medicines3030021

**Published:** 2016-08-05

**Authors:** Narda G. Robinson

**Affiliations:** Department of Clinical Sciences, Colorado State University, Fort Collins, CO 80526, USA; narda@onehealthsim.org; Tel.: +1-970-443-3588

**Keywords:** acupuncture, education, evidence-based

## Abstract

As enthusiasm for alternatives to pharmaceuticals and surgery grows, healthcare consumers are turning increasingly to physical medicine modalities such as acupuncture. However, they may encounter obstacles in accessing acupuncture due to several reasons, such as the inability to locate a suitable practitioner, insufficient reimbursement for treatment, or difficulty gaining a referral due to perceived lack of evidence or scientific rigor by specialists. Claims made about a range of treatment paradigms outstrip evidence and students in acupuncture courses are thus led to believe that the approaches they learn are effective and clinically meaningful. Critical inquiry and critical analysis of techniques taught are often omitted, leading to unquestioning acceptance, adoption, and implementation into practice of approaches that may or may not be rational and effective. Acupuncture education for both licensed physicians (DOs and MDs) and non-physicians needs to include science (i.e., explanation of its effects based on contemporary explanations of biological processes), evidence, and critical thinking. Erroneous notions concerning its mechanisms such as moving “stuck Qi (Chi)” or “energy” with needles and that this energy stagnates at specific, tiny locations on the body called acupuncture points invite errors in methodologic design. For example, researchers may select sham and verum point locations that overlap considerably in their neural connections, leading to nonsignificant differences between the two interventions. Furthermore, attributing the effects of acupuncture to metaphorical and arcane views of physiology limits both acceptance and validation of acupuncture in both research and clinical settings. Finally, the content and quality of education and clinical exposure across acupuncture programs varies widely, with currently no minimum basic educational requirements in a scientific methodology. Considering the pressures mounting on clinicians to practice in an evidence-based and scientific manner that also demonstrates cost-effectiveness, acupuncture schools and continuing medical education (CME) courses should provide their students a strong foundation in rational approaches supported by research.

## 1. Introduction

Today, acupuncture, especially in the West, spans a broad continuum [1]. At one pole sit the strictly scientific practitioners, i.e., those that eschew the myriad metaphors and metaphysical, mystical mechanisms so long associated with this form of medicine. At the other end reside acupuncturists content to view their practice as the manipulation and management of subtle energy circuits, relying on subjective observations of the patient that then determine both diagnosis and treatment.

The majority of physician acupuncturists likely land somewhere between these two ends of the spectrum, practicing a panoply of techniques that have greater and lesser degrees of scientific backing. Acupuncture coursework may focus more fully on one or a few approaches, whether neuroanatomic or myofascial trigger point therapy (i.e., the scientific methods) or Traditional Chinese Medicine, microsystems, Five Phases (or Five Elements), and more (largely abstract, metaphorical, irrational, or otherwise unscientific approaches). What physician acupuncturists learn in a given course stems more from the intellectual and philosophical proclivities of the course director(s) rather than agreed-upon standards profession-wide. Thus, in North America, acupuncture education for physicians has existed, for the most part, on its own in CME programs [2] or acupuncture schools [3] apart from coursework consisting of the “mainstream” of medicine, leading to, in some cases, markedly different standards and expectations for curricula and graduates.

With course content depending solely on the determination of each individual course director, students in that program sign up not only to learn “acupuncture” but in effect are brought into the fold of one or more systems with premises they may or may not agree. That is, should a medical practitioner find the approaches of neuroanatomic acupuncture and trigger point therapy more attractive because they rest on biomedical principles, that individual needs to select carefully a course of study that aligns with those ideals. Otherwise, one could find oneself in a program that espouses what he or she might consider pseudoscience, i.e., explaining acupuncture mechanisms metaphysically rather than through analysis of the actual, tangible, readily identifiable structures involved [4,5]. This divergence sets up a dichotomy within the profession: some practice in accordance with scientific principles and others based on abstract explanations. However, even if a physician seeks a purely scientific acupuncture education, s/he must learn a broad array of philosophies and techniques, some based on abstract and metaphorical approaches, in order to become board certified in medical acupuncture, as reflected in the recommended readings for the American Board of Medical Acupuncture board certification examination [6].

Whereas physicians typically spend years in medical school and postgraduate programs and then add acupuncture as CME, certified acupuncturists typically spend years in school learning acupuncture and herbal treatment medicine [7]. However, most programs that train certified or licensed acupuncturists (L.Ac.’s) teach that acupuncture works, at least in part, by moving energies and eliminating “pathogenic factors” such as metaphorical wind, damp, heat, and cold. Their qualifying examination (i.e., that offered by the National Certification Commission for Acupuncture and Oriental Medicine or NCCAOM) reflects this. It also includes testing on clinical examination methods [8] such as tongue and pulse diagnosis that have low interrater reliability and little proven validity as diagnostic tools [9,10]. Considering that healthcare decisions made by acupuncturists rely heavily on the subjective assessment by the practitioner, relying on arcane and unproven methods to differentiate between health and disease puts patients at risk medically and financially if limited resources lead to ineffective or harmful treatments.

While current medical diagnostic approaches can also have some subjectivity and inter-rater differences, the assessments made involve actual anatomical structures such as trigger points [11] that are palpated or otherwise analyzed, not abstract inferences concerning organ function based on the appearance of the tongue or the quality of the pulse.

## 2. Overview

In postgraduate medicine programs, one assumes that academic educators regularly undergo self-examination and question whether requirements for postgraduate training, licensure, and specialty education are meeting the intended objectives [12]. As techniques develop and evidence accrues, needs for certain types of knowledge or skills may change. Furthermore, in most other courses of study, a student may safely assume that the premises and content within a CME course are valid, tested, and reliable. This does not necessarily apply to acupuncture, given the wide variation in content one might find, ranging from a discussion of the neuroanatomy and neurophysiology of acupuncture to what some might call “superstition”, i.e., processes that contradict natural science.

In such instances, uncertainty about the veracity of the techniques taught put the learner, no matter how earnest, at a clear disadvantage. She or he may have mistakenly assumed that by definition a continuing medical education program, especially one approved for CME credits, would be quality controlled for rational content and substantiation. While it is wise to think critically about any course content, most physicians attending conventional CME courses do not find themselves needing to do further research on their own in order to determine whether what they learned defied rational explanation and evidential support. With acupuncture, they do, at least for now where training programs offer such widely divergent methodologies.

Currently, patients seeking care even from board-certified physician acupuncturists could receive treatment ranging from rational and evidence-informed methodologies (such as neuroanatomic acupuncture or trigger point therapy) or approaches that have no scientific basis and little to no evidence of effectiveness. This uncertainty places them in a vulnerable position, i.e., assuming and expecting a level of care in accordance with contemporary biomedicine as well as a fair expectation of a beneficial clinical outcome. Approaches practiced by certified acupuncturists typically involve Traditional Chinese Medicine (TCM) philosophies and practices—also unscientific. Remaining unaware and, in some cases, uninterested in the actual anatomy of acupuncture and its underlying physiologic mechanisms of needling and related techniques hinders one’s ability justify point selection and defend treatment techniques, even though this information is available [4].

Even if the practitioner believes that she or he is moving mysterious energies, acupuncture often has physiologic effects, thereby reaffirming his or her belief that what was taught was real, but that faith is misplaced. For example, microsystems approaches such as scalp, hand, and ear acupuncture have many followers and some research, but the premises upon which they rest are fundamentally flawed and unsupported by evidence. No matter what one believes in terms of special “maps” of the body on the surface of the head, hand, or ear, acupuncture stimulation leads to physiologic changes that can be therapeutic. Does that mean that the topography of the ear actually contains an implied inverted fetus somewhere encoded into its tissue? No. It merely indicates that the cranial and upper cervical nerves that supply the ear cause myriad physiologic changes when stimulated.

Despite there being a clear, neuroanatomic description of the neural supply of the ear and reflex connections through the brainstem that explain how auricular point stimulation neuromodulates vagal pathways as well as evidence that does not support the theory of a highly specific functional map on the ear [13], the majority of practitioners of auricular acupuncture cling to unfounded notions that the auricular acupuncture diagrams are accurate and reliable [14]. This leads to inconsistent results in research and in the practice of auricular acupuncture [13]. Unless and until a standardized auricular acupuncture map [15] becomes available and is based on systematic analysis of the physiologic and anatomic outcomes of point stimulation rather than opinion and voting [16], current “ear maps” are no more reliable than other fanciful, imagined maps as in scalp acupuncture [17,18,19], Korean hand acupuncture [20], foot reflexology, and iridology.

Problems plaguing auricular acupuncture, if resolved, could facilitate its integration into conventional settings such as the emergency department [21,22] and drug detoxification settings [23].

### 2.1. Is There a Better Way?

There is another option. Insights into the anatomy and physiology of acupuncture have grown to where there is no longer any need to rely on metaphysical ideations regarding how acupuncture works. Having certification programs institute a minimum basic requirement for scientific content and critical thinking skills, would ensure that all practitioners certified by that group could describe and defend their scientific acupuncture techniques to colleagues, insurers, and institutions. They would also become better researchers and advocates for the profession. As stated by Ning and Lao, “The most critical challenge in acupuncture research is the selection of controls and the design of appropriate sham needling [24].” Designing higher quality studies mandates that the researchers have a clear picture of what constitutes an acupuncture point and how the structures affiliated with that site produce the physiologic effects classically attributed to that site’s activation.

### 2.2. Proposed Curricular Content

In which ways, specifically, could acupuncture schools and CME programs ensure that training protects the public by graduating highly qualified acupuncturists with a strong background in science and evidence that can provide safe, efficacious, and cost-effective health care [25]? What information and strategies should acupuncture providers learn that would give them the ability to discern between rational and irrational approaches, whether in their work as clinicians, researchers, or educators? How can acupuncture education evolve so that training is “based on rigorous scientific evidence and a robust system of quality assurance [25]”?

To begin with, acupuncture students should learn about acupuncture history, become familiar with research milestones, and develop critical thinking skills that will show them how to assess the veracity and validity of acupuncture principles and practices. For example, instead of learning how to perform tongue and pulse “diagnosis” in preparation for determining the diagnosis and treatment approach as in Traditional Chinese Medicine (TCM), students should first learn to how to appropriately question and critique research on these highly unreliable measures. Before moving forward with adopting these skills, they need to understand that neither pulse nor tongue diagnosis has been validated as a reliable method by which to determine health or pathology in a patient [26,27]. At least by recognizing the high level of subjectivity in these tools, acupuncturists have the opportunity to introduce caution in the judgments they make. Treatment follows diagnosis. As such, if the diagnostic techniques are neither reliable nor valid, the veracity of a patient assessment and the effectiveness of treatment come into question.

Acupuncturists also need to become acquainted with Western (scientific) medical acupuncture [26], its rational premise, and methods of diagnosis and treatment that include and rely heavily upon appropriate, well established analyses as well as myofascial palpation.

The anatomical segment of the minimum basic requirement in science would include in-depth study of point anatomy as well as discussions about how the structures at each point relate to the physiologic effects one expects to see based on clinical experience and/or research-based evidence.

For clinical applications, students would learn the central tenets of neuromodulation [28,29] and connective tissue effects [30] as the basis of acupuncture, continue with a critical examination of acupuncture research methodologies and flaws, and proceed into a science-based, evidence-informed review of a scientific approach to the gamut of medical conditions and clinical challenges effectively treated by acupuncture. They would be able to differentiate scientific medical acupuncture from other forms of acupuncture [26].

### 2.3. Encouraging Better-Designed Acupuncture Research

In order to best serve patients and make the best use of healthcare dollars and resources [31], acupuncture practitioners need factual and reliable information on which to base their care and inform their patients or clients of the relative merit of various options. This information comes from rigorous, scientific research. However, the same problems that plague acupuncture practice (i.e., outmoded or imagined mechanisms of action, methods of diagnosis that lack validity and reliability, unsubstantiated premises of treatment) also plague acupuncture research. New standards have been developed [32], but problems remain.

Furthermore, the lack of distance between verum and sham input effects and the inclusion of primitive, folkloric diagnostic methods, lay the acupuncture profession open to criticism from skeptical observers who claim that acupuncture is a “theatrical placebo” [33,34]. Graduates of acupuncture educational programs should be well-equipped to respond to critics that dismiss acupuncture as nothing more than placebo with facts from the latest research [35,36]. Most practitioners of acupuncture, whether MDs, DOs, or LAcs, would not feel confident building a strong, science based defense at this time.

## 3. Conclusions

Requiring a solid foundation in the science and evidence of acupuncture does not make the approach any less of an art. Rather, a clinician who recognizes and addresses the multifaceted nature of neural and connective tissue interrelationships is working with active and anatomically identifiable structures rather than ethereal energies that may not even exist.
